# Targeting IL-17 for pancreatic cancer prevention

**DOI:** 10.18632/oncotarget.2618

**Published:** 2014-10-22

**Authors:** Florencia McAllister, Steven D. Leach

**Affiliations:** Department of Clinical Cancer Prevention, The University of Texas MD Anderson Cancer Center, Houston, TX; The David M. Rubenstein Center for Pancreatic Cancer Research, Memorial Sloan Kettering Cancer Center, New York, NY

It is well known that pancreatic ductal adenocarcinoma (PDAC) has a dismal prognosis, which can be explained by both an inherently aggressive biology and a lack of effective chemotherapeutics. As a result, there is strong rationale for the development of effective preventive interventions in high risk populations. Chronic pancreatitis increases the risk of developing PDAC more than fifteen fold. Using selective activation of oncogenic Kras specifically in the murine pancreatic epithelium, multiple groups have shown that murine chronic pancreatitis, induced by cerulein, accelerates the development of pancreatic intraepithelial neoplasia (PanIN) and PDAC. An improved understanding of the cellular and soluble mediators responsible for this effect would have a significant impact on the development of preventive strategies for this disease.

Our group has recently reported that IL-17-producing-CD4+T cells (T_H_17) and IL-17 producing-gamma-delta T cells are increased in the dense fibro-inflammatory stroma surrounding Kras-induced murine PanINs. The abundance of these IL-17 producing cells is also synergistically augmented in the context of associated chronic pancreatitis [1]. In the same mouse model, neutralization of the IL-17 pathway using monoclonal antibodies against IL-17 isoforms and the IL-17 receptor (IL-17RA) effectively prevents PanIN initiation and progression. Overexpression of the IL-17RA has been detected in murine PanINs, and IL-17-dependent gene expression signatures have been detected in PanIN epithelial cells, suggesting the existence of a functional hematopoietic-to-epithelial IL-17 signaling axis as a potent driver of PanIN formation. The IL-17RA has also been detected in human PanINs, and immune cells expressing RORγt, a key transcription factor required for the differentiation of the T_H_17 lineage, have been detected infiltrating human PanINs, emphasizing the translational relevance of the findings [1].

IL-17-producing T Helper cells (T_H_17) play an active role in chronic inflammation but their role in tumorigenesis remains controversial. On one hand, IL-17 has been associated with migration, invasion, recruitment of myeloid derived suppressor cells and the induction of angiogenic and anti-apoptotic factors. On the other hand, T_H_17 cells have been reported to mediate anti-tumor effects by promoting CTLs activities, MHC antigen expression, and production of IFN-γ. However, most of the anti-tumor effects reported have been described in the setting of advanced cancer.

Recently, the International Cancer of the Pancreas Screening (CAPS) Consortium summit recommended that pancreatic cancer screening programs should be implemented on well-defined high risk groups, with the goal of detecting and treating resectable T1N0M0 PDAC and high-grade dysplastic precursor lesions. Recommended initial screening modalities include endoscopic ultrasonography (EUS) and/or MRI/magnetic resonance cholangiopancreatography. We would speculate that immune-prevention could be ideally implemented on patients enrolled into these screening programs.

A recent large case-control study showed that a daily aspirin regimen may reduce the risk of developing pancreatic cancer in the general population, highlighting the importance of inflammation in pancreatic tumor initiation and progression. However, we would anticipate that targeted immune-preventive interventions may have superior efficacy in high risk patients. A variety of antibodies targeting the IL-17 signaling pathway have been under development for autoimmune diseases during the past decade. Ixekizumab and secukinumab, monoclonal antibodies against IL-17, and brodalumab, a human monoclonal antibody directed against the IL-17 receptor (IL-17RA), have shown efficacy in randomized phase II trials for psoriasis and are currently being tested in phase III clinical trials. In 2013, the FDA has approved the use of ustekinumab, an antibody that blocks the p40 common subunit of IL-12 and IL-23, for use in moderate-to-severe psoriatic arthritis and Crohn’s disease. Finally, monoclonal antibodies that specifically block the p19 subunit of IL-23 (not shared by IL-12), are currently under development and expected to have similar efficacy to IL-12 with even less adverse effects.

Based on preclinical data documenting a requisite role for IL-17 in PanIN progression, specific targeting of the IL-17 pathway with monoclonal antibodies might represent an effective strategy for pancreatic cancer prevention. Before moving forward with such studies, however, additional work is required to better determine the influence of IL-17 on already established pancreatic cancers, as well as the role that IL-17 might play in in the context of immunotherapeutic interventions. By determining how IL-17 influences the entire spectrum of pancreatic cancer initiation and progression, the future of IL-17 targeting as a means of pancreatic cancer chemoprevention will become clear.
